# New Drugs and Preparations

**Published:** 1895-02-02

**Authors:** 


### NEW DRUGS AND PREPARATIONS.

[We shall be glad to receive, at our Office, 428, Strand, London, W.C., from the manufacturers, specimens of all new preparations and drugs which may be brought out from time to time.]

### THE ACTION OF SOME HOMOLOGUES OF QUININE.

Grimaux has communicated to the Academy of Sciences (June 11th, 1894) the results of a research on bodies allied to quinine, which he has made in conjunction with Drs. Laborde and Bourru.

Cupreine, methyl-cupreine (quinine), ethyl-cupreine, propyl-cupreine, and amyl-cupreine were all examined, the new basis being formed by introducing the various alcoholic radicals into the original alkaloid cupreine.

Methyl-cupreine (quinine) was found to be about twice as poisonous as cupreine, which it closely resembles in action; ethyl-cupreine, again, is still more toxic, and propyl-cupreine still more, the toxicity and power of reducing temperature becoming greater as the higher homologous radicals are introduced into cupreine. Therapeutically, the same results were borne out. Cupreine was given in various forms of malarial fever in seven to twenty-two grain doses, The smallest dose in which it shows any action is about fifteen grains, and it is not nearly so powerful an antiperiodic as quinine is. Ethyl-cupreine was given as the basic sulphate in seven to eighteen grain doses, and always showed itself to be a powerful antiperiodic, succeeding in several cases where quinine had already failed. In the above doses it never induced any toxic symptoms whatever in the patients. Propyl-cupreine was also administered as the basic sulphate, but never in doses above nine grains. It is an excellent antiperiodic also, as well as antipyretic in about seven grain doses. It, however, is apt to cause singing in the ears, vertigo, nausea, and general depression. The authors think that ethyl-cupreine (or quin-ethyline as it may also be called) will prove to be superior in practical value to quinine.

